# A Design of Wireless Sensor Networks for a Power Quality Monitoring System

**DOI:** 10.3390/s101109712

**Published:** 2010-11-01

**Authors:** Yujin Lim, Hak-Man Kim, Sanggil Kang

**Affiliations:** 1 Department of Information Media, University of Suwon/San 2-2, Wau-ri, Bongdam-eup, Hwaseong-si, Gyeonggi-do, 445-743, Korea; E-Mail: yujin@suwon.ac.kr; 2 Department of Electrical Engineering, University of Incheon/12-1, Sondo-dong, Yeonsu-gu, Incheon, 406-840, Korea; 3 Computer Science and Information Engineering, Inha University/253, Yonghyun-dong, Nam-gu, Incheon, 402-751, Korea; E-Mail: sgkang@inha.ac.kr

**Keywords:** wireless sensor network, electric distribution system, power quality, tree-based protocol, data forwarding protocol

## Abstract

Power grids deal with the business of generation, transmission, and distribution of electric power. Recently, interest in power quality in electrical distribution systems has increased rapidly. In Korea, the communication network to deliver voltage, current, and temperature measurements gathered from pole transformers to remote monitoring centers employs cellular mobile technology. Due to high cost of the cellular mobile technology, power quality monitoring measurements are limited and data gathering intervals are large. This causes difficulties in providing the power quality monitoring service. To alleviate the problems, in this paper we present a communication infrastructure to provide low cost, reliable data delivery. The communication infrastructure consists of wired connections between substations and monitoring centers, and wireless connections between pole transformers and substations. For the wireless connection, we employ a wireless sensor network and design its corresponding data forwarding protocol to improve the quality of data delivery. For the design, we adopt a tree-based data forwarding protocol in order to customize the distribution pattern of the power quality information. We verify the performance of the proposed data forwarding protocol quantitatively using the NS-2 network simulator.

## 1. Introduction

**Figure 1.**
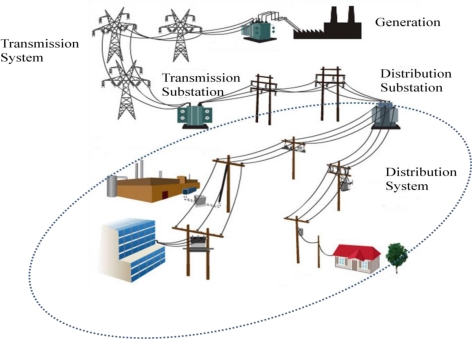
An example of a power grid.

Power grids involve the generation, transmission and distribution of electric power. Figure 1 shows an example of the configuration of a power grid. The electrical distribution system delivers electric power through feeders and pole transformers from distribution substations to end users such as houses, office buildings, and factories. Power quality is any power problem manifested in voltage, current, or frequency deviations, that results in failure or malfunctioning of the customer equipment [1]. It is one of the most important issues in electrical distribution systems.

Korea Electric Power Corporation (KEPCO) [2] in Korea has monitored basic electrical quantities such as voltage and current from major pole transformers using their temperature. At present and in order to reduce the transmission costs, the information gathered from major pole transformers is transmitted every 30 minutes to a remote monitoring center over the cellular communication network utilized by KEPCO. KEPCO is trying to expand the power quality monitoring system to gather and transmit data towards a monitoring center with low communication costs and at shorter transmission intervals. The system gathers the information about power quality features such as harmonics, voltage sags, and voltage swells. The gathered information is transmitted to accomplish various tasks such as evaluating the current status of power quality, finding places where power quality problems are occurring, and planning measures to improve power quality.

Various issues such as measurements, controls, databases, and communications must be considered for expanding the power quality monitoring system. We focus on designing an efficient communication network in the electrical distribution system (EDS). Wireless multi-hop communication paradigm is often employed to construct EDS to reduce the deployment and management costs. While wireless sensor networks (WSNs) have been widely studied in the abstract [3–9], to our best knowledge not many studies have focused on building EDS.

A WSN is a system composed of numerous computing and sensing devices distributed within an environment to be monitored. In the past decades, WSNs have been applied to autonomous use of computing, sensing, and wireless communication devices for both scientific and commercial purposes [10,11]. For example, ZebraNet [12] has been developed for wildlife tracking. CitySense [13] has been devised to report weather and air pollution. The Sensormap portal [14] has been developed for genetic monitoring services. Research attention has been increasingly paid to designing customized systems like the above to satisfy application-dependent service requirements.

Our goal was to design a reliable WSN for a power quality monitoring system to provide high data delivery quality with the least cost of communication system installation and maintenance. EDS delivers power quality information generated by pole transformers to remote monitoring centers in residential divisions. Usually, the power quality information is periodically measured, gathered, and transmitted to the monitoring centers. Once power quality measured at a pole transformer is out of a normal range, an alarm message with detailed contents is promptly sent in the event based manner. To improve the performance in terms of the data delivery rate and the distribution delay of the power quality data, we customize our communication protocol for the distribution pattern of the power quality information. We also examine the performance tradeoffs between the data delivery rate and the distribution delay of the power quality data.

The remainder of this paper is structured as follows. Section 2 describes our system architecture and design goals. Section 3 explains our communication infrastructure and discusses the proposed tree-based data forwarding protocol. Following this, we verify the designed system by NS-2 simulations in Section 4. Finally, Section 5 summarizes our results, discusses our future plans, and offers conclusions.

## 2. Electrical Distribution System

### 2.1. System Architecture

The EDS in Seoul, Korea [2] has tens of thousands of pole transformers ranging widely over hundreds of square kilometers. A monitoring center in a residential division of the city is a data collection point which gathers the power quality information from scattered pole transformers deployed over the city.

**Figure 2.**
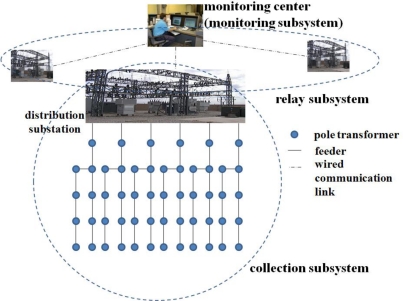
Electrical distribution system.

The distribution network for the EDS consists of three subsystems as shown in Figure 2: A collection subsystem, a relay subsystem, and a monitoring subsystem. The collection subsystem is composed of several distribution substations (hereafter, the term ‘substation’ is exchangeable with ‘distribution substation’). Each substation is connected to several feeders. Each feeder collects the power quality data from hundreds of pole transformers and delivers them to the substation. The relay subsystem is responsible for delivering the data gathered by the substations to the monitoring subsystem. The monitoring center in the monitoring subsystem processes the power quality data to recognize the current status of situations and takes appropriate actions based on the assessed situation [15,16].

### 2.2. Design Goals

Despite the fact that power quality monitoring in EDS is becoming more and more important, the performance of existing systems is not sufficient to provide expanded power quality monitoring services. The system has a quite high communication cost of installation and maintenance. In the systems, a major pole transformer periodically gathers data sensed from pole transformers within its coverage using single-hop or multi-hop communication. The major pole transformer sends the gathered data towards the monitoring center through the cellular communication network every 30 minutes. The limitations of the monitoring system are as follows: first, since the data is only transmitted every 30 minutes due to the high communication cost, the power quality data cannot be delivered in a timely fashion to the remote monitoring centers. Second, it is difficult to monitor power quality using only the basic electric quantities such as voltage, current, and temperature of pole transformers. Finally, data collection is severely limited for a specific small area.

To overcome the limitations, we have designed a communications system to provide data delivery service in a timely and reliable manner. The design goals of our system are as follows:

♦ The power quality data is measured every one second.
♦ The measured data is transmitted to the monitoring center every transmission interval.
♦ The system is operated over a medium or large-size city. We plan to deploy our system at the area which is covered by a residential division.
♦ For the usual data, a high data delivery ratio is important within the acceptable transmission delay.
♦ For the alarm data, the distribution delay of the power quality data is important as well as the data delivery ratio.
♦ The use of the current off-the-shelf radio interfaces is recommended.

## 3. Design of a Wireless Sensor Network for a Power Quality Monitoring System

### 3.1. Design of Communication Infrastructure

In this subsection, we design a communication infrastructure for EDS power quality delivery as seen in Figure 2. The communication infrastructure consists of two parts; wired for the relay subsystem and wireless for the collection subsystem. The relay subsystem forwards data from substations to the monitoring subsystem via wired infrastructure due to the long distances between the relay subsystems and the monitoring subsystems. In the collection subsystem, pole transformers have been deployed sparsely at distances of hundreds of meters. We employ a WSN to construct the collection subsystem, in order to reduce the deployment and management cost. Since substations in the relay subsystem are connected to the monitoring center through a high-speed wired network, the communication between them is highly reliable. Thus the problem of data delivery in EDS is the same as the data delivery problem at the collection subsystem.

The collection subsystem can be implemented by using the IEEE 802.15.4 [17] or IEEE 802.11b standards [18]. A wireless personal area network (WPAN) based on the IEEE 802.15.4 standard has been used due to the short communication range (e.g., dozens of meters) and the extra low power consumption of a communication devices, in spite of its low data rate. However, in EDS, pole transformer communication devices have been deployed at distances of hundreds of meters. Energy issues do not arise because the EDS is assumed to have the support of power lines. We use Wi-Fi (IEEE 802.11b) radio modules. The advantages of these modules are high data throughput (e.g., 11 Mbps) and the ability to abstract wireless communication details including collision detection and avoidance and error detection.

### 3.2. Design of Wireless Sensor Node

**Figure 3.**
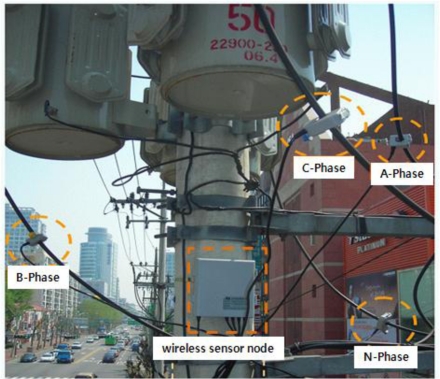
Wireless sensor node design on a pole transformer.

**Figure 4.**
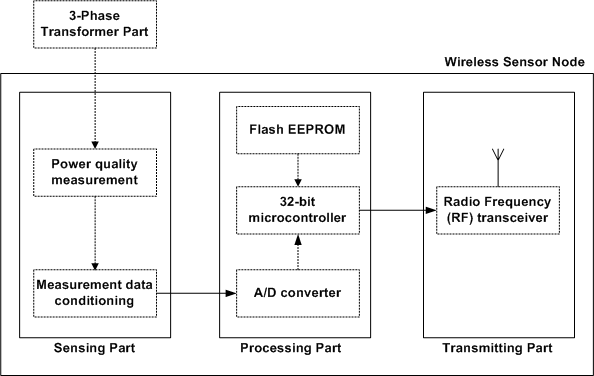
Block diagram of a wireless sensor node.

Figure 3 shows the wireless sensor node design on a pole transformer. The block diagram in Figure 4 illustrates the components, consisting of wireless sensor node, and their interactions with one another. Through the 3-phase transformer part, power quality is measured periodically. Measurements are carried out once every second, which is sufficient to provide the power quality monitoring service. Assuming that we store 3,600 data per hour, each hour requires a little over 500 Kbytes of space. This implies that 640 Kbytes of flash RAM are sufficient to store the data. The microprocessor has a number of functions: processing data collection from the sensing part, interfacing the data to the physical radio layer, and managing the radio network protocol. We use the OEM wireless Ethernet (IEEE 802.11b) module with a range of about 500 meters for peer transfers among neighboring pole transformers.

### 3.3. Design of Power Quality Information

Power quality is any power problem manifested in voltage, current, or frequency deviations, that results in failure or malfunctioning of the customer equipment. Power quality information includes harmonics, voltage sags, voltage swells, unbalance of network voltage, electric switching device load effects, and so on. Our power quality system monitors the first three information items. Table 1 shows the information per phase gathered at each pole transformer.

### 3.4. Design of Data Forwarding Protocol

Since not every transformer is placed within the communication range of the monitoring center, data cannot be sent directly. Instead, it has to hop its way towards the monitoring center using other transformers as intermediate hops. In EDS, all pole transformers can be data sources, while the monitoring center alone is a data sink. In addition, the network topology in EDS is stationary. We design the data forwarding protocol for the collection subsystem. Since multi-hop and many-to-one nature of data flows, we come up with a new tree-based data forwarding protocol for delivery of the power quality information from each pole transformer (hereafter, we will use the term ‘node’) to a monitoring center.

One of cost factors in constructing the least cost path from a node to the substation is the number of hops between the node and the substation. At the network initialization stage, the substation floods a PROBE message over the entire network so that each node in the network can infer the minimum number of hops from the substation to itself. Thereafter, the substation floods a PROBE message periodically so that nodes can update their hop distance from the substation. The path cost is used in constructing the tree branch between the substation and one node.

The substation starts the tree construction procedure by sending a PROBE message to its neighboring nodes via one-hop flooding. In the PROBE message, the path cost from the substation to the node having sent the message is recorded. When a node receives a PROBE message, it compares the path cost stored in the message with its path cost. If the path cost maintained locally at the node is less than that in the PROBE message, the node ignores the message. Otherwise, the node updates its path cost and configures the node having sent the PROBE message as its parent node. Once the node newly determines its parent node, it sends a PROBE message with updated path cost to its neighboring nodes via the one-hop flooding. This process continues until the node which has no child node receives a PROBE message.

Each node on the tree maintains the soft states on its parent node and child nodes in its forwarding table. A node periodically sends a HELLO message including its parent node ID to its neighboring node as its heartbeat. When a node receives a HELLO message, it checks whether the sender of the message is one of the parent node and child nodes. If it is, the node having received the message updates the soft state on the node having sent. Otherwise, if the node having received the HELLO message is the parent node of the node having sent, it adds the node having sent to a list of its child nodes.

**Figure 5.**
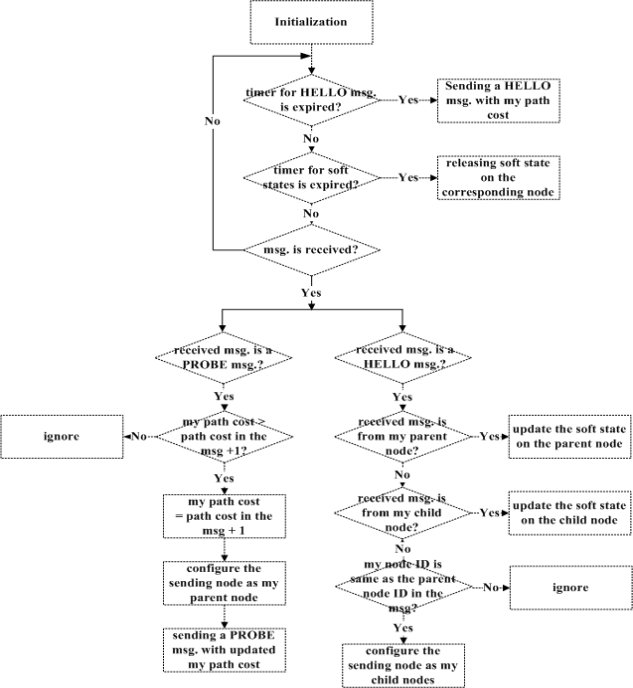
Tree construction procedure.

If a node or the wireless link to the node fails, its parent node and child nodes do not receive any HELLO messages from the node for a given amount of time. Thus, the parent node and child nodes of the failed node release the soft state on the node. The node detecting the failure of its parent node tries to repair the broken tree branch by sending a REPAIR message to its neighboring nodes via the one-hop flooding. Once a neighboring node receives the REPAIR message, it responds with a REPAIR_ACK message having its path cost. Then, the node having sent the REPAIR message receives the REPAIR_ACK message(s) and it selects the node having the least path cost as its parent node. The detailed procedure of the tree construction is depicted in Figure 5.

Once the data forwarding tree is constructed, the power quality data is delivered to the monitoring center through the tree. Whenever a node has data to send, periodically or in the event-based manner, the node transmits the data to its parent node. The parent node checks whether the sender is one of its child nodes. If it is, the parent node forwards the data to its upstream node towards the monitoring center. This forwarding process continues until the monitoring center receives the power quality information.

## 4. Performance Evaluation

In this section, we will describe our simulation environment for our protocol evaluation. To evaluate the performance of the proposed data forwarding protocol quantitatively, we use NS-2 network simulator [19]. The collection subsystem delivers the power quality data to the substation in a timely and reliable manner. We choose the quality of data delivery in terms of the data delivery ratio and the distribution delay of the power quality data as performance metrics.

The collection subsystem is made up of one substation and six feeders. Each feeder collects the power quality data from 300 pole transformers placed in a random pattern. In other words, a total of 1,800 nodes are deployed in the network. The distance between two neighboring pole transformers is set to around 200–300 meters. The transmission radius of a node (a communicating device on the pole transformer) is 500 m. We use the shadowing model in [20] to model a radio propagation environment. IEEE 802.11b is used as the MAC layer in our experiments and its data rate is 11 Mbps. Each node measures the power quality data every one second and transmits the measured data towards the substation at every transmission interval, which ranges from one second to 60 seconds. In our collection subsystem, packets travel along a chain of intermediate nodes towards the substation except for branch nodes. Since radios can interfere with each other beyond the range at which they can communicate successfully, the utilization of a chain of nodes is quite low compared to the maximum throughput [21,22]. As the offered load increases beyond this maximum utilization, the chain throughput drops sharply, so the offered load should be controlled. We vary the offered load to achieve the maximum performance in terms of the quality of data delivery by adjusting the data transmission interval of nodes.

The traffic pattern generated by each node is the constant bit rate (CBR). The size of a CBR data packet is set to 1,500 bytes and the total simulation time to 3,600 seconds. Since the RTS/CTS/ACK exchange adds significant overhead in the IEEE 802.11 based network, the data throughput with 1,500-byte data packet is higher than that with 64-byte or 500-byte packet [21]. Thus we employ a 1,500-byte length data packet to forward the aggregated power quality data instead of multiple short packets.

Since the packet drop probability in a wireless multi-hop communication environment increases with the number of hops [22], in the simulation we generated three different topologies with 300 nodes: a 30 hops-sized network, a 50 hops-sized network, and a 70 hops-sized network. The 30 hops-sized network represents the longest hop distance between a substation and a node is 30 hops in a feeder.

**Figure 6.**
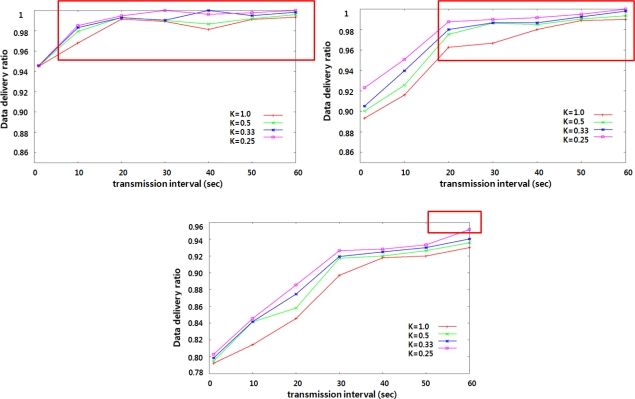
Data delivery ratio with increasing the transmission interval. **(a)** 30 hops-sized network topology **(b)** 50 hops-sized network topology **(c)** 70 hops-sized network topology.

Figure 6 shows the data delivery ratio when the transmission interval of the power quality data is varied from one second to 60 seconds. The data delivery ratio means the average ratio of the number of data messages received by the substation to the total number of data message sent by all data sources. As mentioned earlier, the power quality data is measured every one second and the measured data is aggregated and transmitted every transmission interval. The value of k in each legend of the figure represents the rate of data source in the network. For example, k = 0.5 represents that 900 nodes (50 percent of all 1,800 nodes) measure the power quality data. The power quality data measured at a pole transformer is likely to be similar to the data measured at its geographically adjacent neighboring pole transformer. Thus, we can control the rate k to achieve the maximum performance as well as the transmission interval. In the figure, over 95 percent of the data delivery ratio is obtained for the conditions: the transmission interval is greater than one second in 30 hops-sized network, than 10 seconds in 50 hops-sized network. It is greater than 50 seconds and the value of k is smaller than 0.33 in 70 hops-sized network. As the transmission interval increases or the value of k decreases, the data delivery ratio increases.

In general, the collision domain size is a major consideration to determine the effective throughput. However, since pole transformers stand in a row in our system, the power quality data is forwarded in a peer-to-peer manner except at branch nodes with more than one child node. The collision domain size is three. As shown in the figure, the effective throughput is acceptable for providing the desired power quality monitoring service.

**Figure 7.**
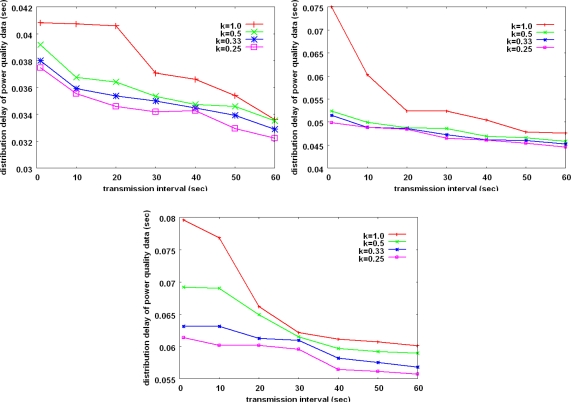
Distribution delay of power quality data with increasing the transmission interval. **(a)** 30 hops-sized network topology **(b)** 50 hops-sized network topology **(c)** 70 hops-sized network topology.

Figure 7 shows the distribution delay of the power quality data when the transmission interval of the data is varied. The distribution delay of the power quality data represents the average time from when a data source sends its power quality data until the substation receives the data.

Only the cases of the substation successfully receiving data are counted in. It is well known that the transmission delay in a wireless *ad hoc* network increases as the network diameter increases [23]. Besides, the offered load which can be controlled by the transmission interval and the value of k affects on the transmission delay. However, as shown in the figure, the distribution delay between a pole transformer and the substation is less than 0.1 seconds. The distribution delay is an acceptable range to provide the monitoring service.

**Figure 8.**
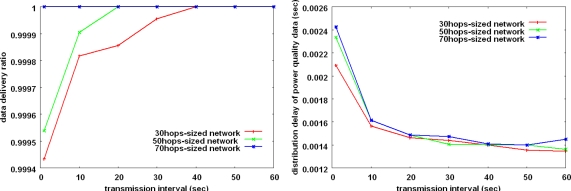
Performance in environment around the substation. **(a)** data delivery ratio **(b)** distribution delay of power quality data.

As we can see in Figure 2, the last hop from six feeders to the substation in the collection subsystem can be the performance bottleneck because all data are gathered at a single collecting point (substation). We have experimented with the performance on the last hop in Figure 8. The value of k is set to 1.0. Contrary to our concern, the bottleneck problem does not occur in the last hop. It is because the collision domain size is still small and the data is not concentrated to the substation simultaneously due to the relatively large data measurement interval and transmission interval.

## 5. Conclusions

We have designed a wireless sensor network for a power quality monitoring system. The contributions of this paper are stated as follows:

1. Our communication infrastructure reduces the cost of network installation and maintenance.
2. Our wireless sensor network delivers rich quantities of data for providing the power quality monitoring service.
3. Our data forwarding protocol provides a data delivery service in a timely and reliable manner.
4. Our experiment shows the feasibility of the designed system by evaluating the performance of our data forwarding protocol using a NS-2 network simulator.

In our work, we did not consider the case that a node is lost or the network is congested in the in-line and multi-hop architecture. It is an important matter to improve the system reliability. As a further work, we will solve the problem using an artificial intelligence technique such as neural network and Bayesian network.

## Figures and Tables

**Table 1.** Power quality information in our system.

Item Byte	ID 2	Temp 1	Hz 1	V_RMS_ 2
Item Byte	I_RMS_ 2	V_DC_ 2	I_DC_ 2	PF 1
Item Byte	V_THD_ 1	I_THD_ 1	V_CF_ 1	I_CF_ 1
Item Byte	V_HF_ Noise 1	I_HF_ Noise 1	Event TY 1	Event TM 2
Item Byte	Event VAL 2	HAR 0∼21 22		SPR 4

ID: identifier of a pole transformer

PF: power factor

Temp: the temperature of a pole transformer

Hz: frequency

V_RMS_: RMS value of voltage

I_RMS_: RMS value of current

V_DC_: DC offset voltage I_DC_: dc offset current

V_THD_: voltage total harmonic distortion

I_THD_: current total harmonic distortion

V_CF_: voltage crest factor I_CF_: current crest factor

V_HF_ Noise: voltage high frequency noise

I_HF_ Noise: current high frequency noise

Event TY: event type Event TM: event time

Event VAL: event value

HAR 0∼21: from fundamental frequency to 21th harmonics

SPR: spare bytes for additional information in the future
